# Repetitive mild traumatic brain injury alters diurnal locomotor activity and response to the light change in mice

**DOI:** 10.1038/s41598-019-50513-5

**Published:** 2019-10-01

**Authors:** Yu-Syuan Wang, Wei Hsieh, Jia-Ru Chung, Tsuo-Hung Lan, Yun Wang

**Affiliations:** 1 Center for Neuropsychiatric Research, National Health Research Institutes, Zhunan, Taiwan; 2 Department of Psychiatry, Taichung Veteran General Hospital, Taichung, Taiwan; 3 Department of Psychiatry, School of Medicine, National Yang-Ming University, Taipei, Taiwan

**Keywords:** Circadian rhythms and sleep, Diseases of the nervous system

## Abstract

Mild traumatic brain injury (mTBI) is a common cause of brain damage with a high incidence of multiple mTBIs found among athletes and soldiers. The purpose of this study is to examine the diurnal behavioral changes after multiple mTBIs. Adult mice were anesthetized; mTBI was conducted by dropping a 30-g weight to the right temporal skull once (mTBI1) or three times (mTBI3) over 3-week. Open-field motor behavior was recorded for 3 days after the last mTBI. In the first 4-hour exploratory phase, mTBI1 or mTBI3 equally reduced locomotor activity. A significant reduction of locomotor activity was found in the dark cycle between 4–72 hour in mTBI1 or mTBI3 mice; higher motor activity was seen after mTBI3 compared to mTBI1. In the light cycle, mTBI3 mice demonstrated an earlier immobilization followed by hyperactivity. The response to light change significantly correlated with the number of impacts. The IBA1 and BAX protein levels were equally increased in the lesioned cortex after mTBI1 and mTBI3. mTBI3 selectively upregulated the expression of circadian clock gene Per1 in hypothalamus and hippocampus as well as iNOS expression in the lesioned side cortex. Our data suggest multiple mTBIs alter diurnal locomotor activity and response to the change of light, which may involve Per1 expression in the lesioned brain.

## Introduction

Traumatic brain injury (TBI) has become an increasingly common cause of brain damage. A recent report estimated the global incidence of TBI is 0.9% and an estimated 69 million people worldwide suffer TBI each year^1^. Of these, the majorities of TBI are mild (81%) in severity. The common causes of mild TBI (mTBI) include falls, vehicle -related collision, violence, and others. Military operations were often associated with a higher incidence of mTBI among soldiers^2^. In a 2009 study, 22.8% of 3973 soldiers from a US army combat team to Iraq had clinician-confirmed TBI^3^. It has been estimated that about 20% of US service members returning from the Operations Iraqi Freedom and Enduring Freedom had TBI^4^. High incidence of sport-related TBI has also been reported^5,6^.

Multiple symptoms develop after mTBI^6^. In experimental animals, mTBI induces sensory-motor abnormality, motor imbalance, and cognitive deficits^7,8^. These behavior changes are associated with apoptosis and neuroinflammation^9,10^ and can be attenuated by pharmacological agents through apoptosis and inflammation pathways^8^. In clinical patients, headache, dizziness, fatigue, difficulty concentrating, and sleep disturbance are the most common complaints following mTBI^11,12^. mTBI alters the quality of sleep in experimental animals and patients^13–15^. An increase in sleep time was found in mice receiving craniotomy and a moderate and diffuse TBI through midline fluid percussion^15^. These findings support that mTBI induces locomotor /cognitive impairments and sleep disturbance.

Repetitive mTBIs increase the risk of neurodegenerative and psychiatric disorders^16,17^. In primary neuronal culture, multiple mTBIs disrupted neuronal membrane integrity, increased ROS formation, and released glutamate^18^. In adult rats, multiple mTBIs resulted in mild spatial memory deficits and anxiety-like behavior associated with microglia activity in the cortex^19–21^. Multiple mTBIs were also commonly seen in athletes and military patients. A survey of 2552 retired NFL players indicated that 24% of these athletes had >3 concussions during their professional playing career^22^. Cognitive impairment, executive dysfunction, and depression were found associated with repeated head impacts or concussions in the football players^22,23^. Without scientific validation, a “three-strike” rule has been adopted as a guideline for athletes with three concussions to limit brain damage^24^. One report indicated that military patients with multiple mTBI or concussions had significantly more severe insomnia or sleep disturbance than patients with no-mTBIs or a single mTBI^25^. These findings support that multiple mTBIs enhance cognitive impairments and neuroinflammation and prior mTBI is a risk factor for future mTBIs.

The purpose of the present study was to examine the changes in diurnal activity pattern after multiple mTBIs in adult mice. An animal model of closed head mild TBI was used to simulate brain concussion in patients^10,26^. In this model, a 30-g impact was delivered to the temporal region of mice; the injury caused apoptosis and neuroinflammation in brain but no physical signs of damage to the skull^26,27^. The open-field motor behavior was examined only once in each animal after the last mTBI to avoid the confound of adaption. Our data suggest that single or multiple mTBIs acutely suppressed exploratory behavior and locomotor activity during the dark time. Multiple mTBI selectively increased the sensitivity to light and disturbed diurnal locomotor activity.

## Results

### mTBI1 and mTBI3 attenuated exploratory behavior at 0–4 hr after injury

**Figure 1:**
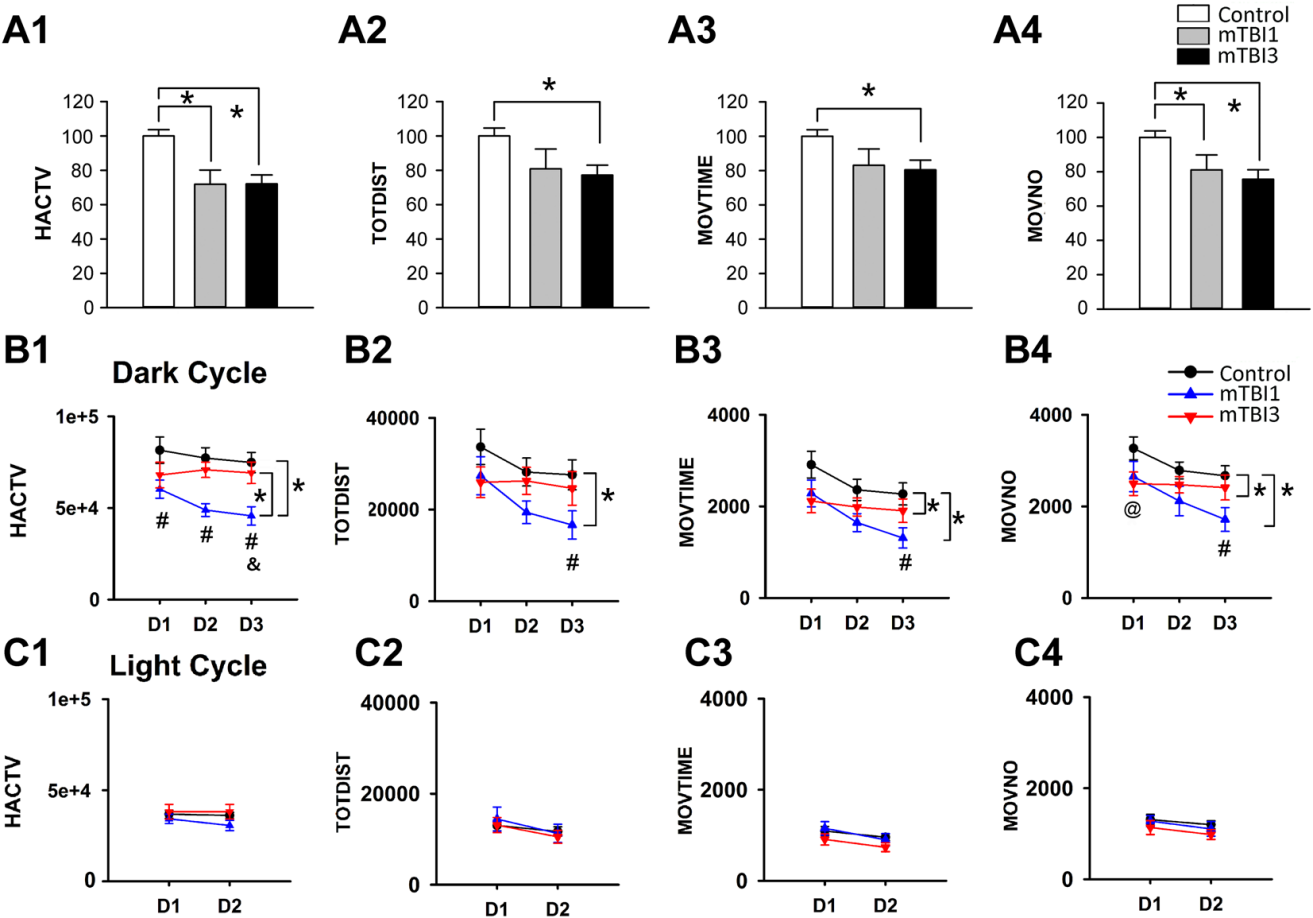
mTBI1 and mTBI3 reduced exploratory activity at 0–4 hr and daily locomotor behavior at 4–72 hr after injury. Mice were individually placed in the infrared activity chambers after the last mTBI or sham procedure, (**A**) The exploratory activity was monitored in the first 4 hr. (**A1**) Horizontal activity (HACTV), (**A2**) total distance traveled (TOTDIST), (**A3**) movement time (MOVTIME), and (**A4**) movement number (MOVNO) were normalized to the mean of no-mTBI control animals. mTBI3 significantly reduced HACTV, TOTDIST, MOVETIME, and MOVNO, compared to no-mTBI. Similarly, mTBI1 significantly reduced HACTV and MOVNO. No difference was found between mTBI1 and mTBI3. (**B**,**C**) Daily light-dark cycle locomotor activity was examined from 4 hr to 72 hr after the last mTBI. (**B**) In the dark cycle, mTBI1 significantly reduced daily 12-hr locomotor activity. A higher locomotor activity was found after mTBI3 compared to mTBI1. ^#^Signficance (by posthoc Fisher test) between mTBI1 and control. ^&^Signficance between mTBI3 and mTBI1. ^@^Significance between mTBI3 and control. (**C**) No difference was found among mTBI1, mTBI3, and control in the daily light cycle. Data are expressed as mean ± SEM. *p < 0.05, (**A**) one way ANOVA, (**B,C**) two-way ANOVA.

A total of 52 mice were used to examine exploratory behavior when animals were habituated in the infrared activity chambers 0–4 hour (hr) after mTBI. Using a 1-Way ANOVA, we found mTBI significantly altered horizontal activity (HACTV, p < 0.001, F_2,49_ = 12.085, Fig. 1A1), total distance traveled (TOTDIST, p = 0.014, F_2,49_ = 4.694, Fig. 1A2), movements time (MOVTIME, p = 0.014, F_2,49_ = 4.696, Fig. 1A3), and number of movements (MOVNO, p = 0.002, F_2,49_ = 7.376, Fig. 1A4). Post hoc Fisher LSD analysis indicates that mTBI3 significantly reduced HACTV (p < 0.001), TOTDIST (p = 0.006), MOVETIME (p = 0.018), and MOVNO (p = 0.002) compared to no-mTBI control (Fig. 1A 1–4). Similarly, mTBI1 significantly reduced HACTV (p = 0.004) and MOVNO (p = 0.032), but not TOTDIST (p = 0.067). No difference was found between mTBI1 and mTBI3 in all these behavioral parameters (HACTV, p = 0.976; TOTDIST p = 0.743; MOVETIME, p = 0.784; and MOVNO, p = 0.555) at 0–4 hr after injury (Fig. 1A 1–4).

### mTBI1 and mTBI3 altered daily locomotor activity between 4 and 72 hr after injury

All animals (n = 52) were housed in the activity chambers for 3 days. The daily behavior activity in the dark (12 hr per day, days 1–3, Fig. 1B) or light (12 hr per day, days 1–2, Fig. 1C) was analyzed after the first 4 hr exploratory period (i.e. 4 to 72 hr after injury). As expected, all animals demonstrated elevated locomotor activity in the dark phase and reduced activity in the light phase (Fig. 1B,C). In the dark cycle, mTBI1 significantly reduced daily HACTV (p < 0.001), TOTDIST (p = 0.020), MOVTIME (p = 0.002), and MOVNO (p = 0.003, Fig. 1B 1–4) compared to no mTBI control (2-way ANOVA). mTBI3 also reduced MOVTIME (p = 0.032) and MOVENO (p = 0.042). Interestingly, a significant increase in HACTV (p = 0.009) was found after mTBI3, compared to mTBI1. The correlation of locomotor activity in the dark cycle and number of mTBIs was next examined by linear regression. The averaged dark cycle locomotor activity did not significantly correlate with the number of impacts (Table 1, HACTV, p = 0.262; TOTDIST, p = 0.33; MOVTIME, p = 0.129; MOVNO, p = 0.118).

**Table 1:** Correlation of the locomotor activity with the number of mTBIs.

		correlation with # mTBIs by linear regression	
		p-value	R-value
Dark cycle activity	HACTV	0.262	0.192
	TOTDIST	0.33	0.167
	MOVTIME	0.129	0.258
	MOVNO	0.118	0.265
Light cycle activity	HACTV	0.692	0.0683
	TOTDIST	0.798	0.0442
	MOVTIME	0.185	0.226
	MOVNO	0.252	0.196
L/D light Sensitivity	HACTV	*0.017	0.397
	TOTDIST	*0.038	0.348
	MOVTIME	0.055	0.323
	MOVNO	*0.04	0.344

Locomotor activity in D1 and D2 were averaged.

In the light cycle, daily locomotor activity was not significantly different among mTBI1, mTBI3, and control (Fig. 1C 1–4, HACTV, p = 0.543; TOTDIST, p = 0.085, MOVTIME, p = 0.063, and MOVNO, p = 0.196). No significant correlation was found between the number of impacts and light cycle locomotor activity (Table 1, HACTV, p = 0.692; TOTDIST, p = 0.798; MOVTIME, p = 0.185; MOVNO, p = 0.252).

### mTBI3 changed locomotor behavior pattern in the light cycle

**Figure 2:**
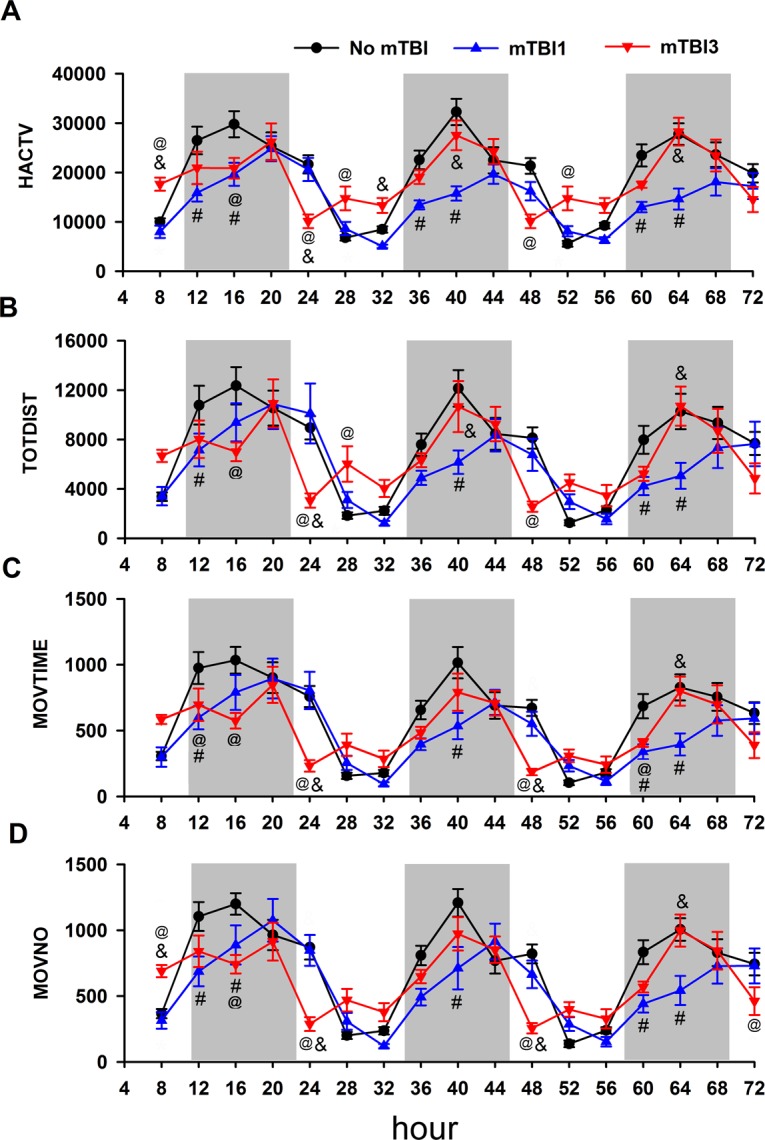
mTBI1 and mTBI3 altered diurnal locomotor activity pattern. (**A**) HACTV, (**B**) TOTDIST, (**C**) MOVTIM, and (**D**) MOVENO were analyzed every 4 hr from 4 to 72 hr after the injury. Open and shadow regions represent the light and dark cycle, respectively. mTBI3 reduced locomotor activity in the early stage of light cycle (i.e. 20–24 hr on **D1** and 44–48 hours on **D2**) while it increased activity in the later stage (24–32 hr and 48–56 hr). In the dark cycle, mTBI1 significantly reduced locomotor activity. mTBI3, compared to mTBI1, significantly increased in locomotor activity in the dark cycle. *p < 0.05 vs control; #p < 0.05 (mTBI3 vs mTBI1). Please also see Supplemental Tables 1 and 2.

Although single or multiple mTBIs did not significantly change the daily 12-hr light cycle locomotor behavior, these injuries differentially altered the pattern of movement when the behavior was analyzed every 4 hours as we previously described^28^ (Fig. 2 and Supplemental Tables 1 and 2). mTBI3 reduced locomotor activity in the early stage of light cycles (i.e. 20–24 hr on D1 and 44–48 hours on D2) while it increased activity in the later stage of light cycles (24–32 hr and 48–56 hr). At 20–24 hr, mTBI3 significantly reduced HACTV (p = 0.007, vs mTBI1; p < 0.001, vs control, Supplemental Table 1), MOVTIM (p = 0.001, vs mTBI1; p = 0.001, vs control; Supplemental Table 2), TOTDIST (p < 0.003, vs mTBI1; p < 0.001, vs control), and MOVNO (p < 0.003 vs mTBI1; p < 0.001, vs control). Similarly, between 44 and 48 hours, mTBI3 significantly reduced HACTV (p < 0.001, vs control; Supplemental Table 1), MOVTIM (p = 0.026 vs mTBI1; p < 0.001, vs control; Supplemental Table 2), TOTDIST (p = 0.051 vs mTBI1; p = 0.006, vs control), and MOVNO (p = 0.013, vs mTBI1; p < 0.001, vs control). In contrast, in the later stage of light cycle, mTBI3 signficantly increased HACTV (p = 0.014, vs control at 24–28 hr; p = 0.033, vs mTBI1 at 28–32 h; p = 0.027, vs control at 48–52 hr; Supplemental Table 1) and TOTDIST (p = 0.020, vs control at 24–28 hr; 2-Way ANOVA, Fig. 2).

### mTBI3 increased locomotor behavior in the dark cycle

Locomotor behavioral was analyzed every 4 hr in the dark cycle. mTBI1, compared to controls, significantly reduced HACTV at 8–12 hr, 12–16 hr, 36–40 hr, 56–60 hr, and 60–64 hr (Fig. 2 and Supplemental Table 1), MOVTIME at 8–12 hr, 12–16 hr, 36–40 hr, 56–60 hr, 60–64 hr; Fig. 2 and Supplemental Table 2), TOTDIST at 36–40 hr (p < 0.001), 56–60 hr (p = 0.038), 60–64 hr (p = 0.004, Fig. 2), and MOVNO at 8–12 hr (p = 0.007), 12–16 hr (p = 0.003), 36–40 hr (p < 0.001), 56–60 hr (p = 0.012), and 60–64 hr (p = 0.002, two way ANOVA, Fig. 2). mTBI3, compared to mTBI1, significantly increased locomotor activity in the dark cycle (HACTV: p = 0.003, 36–40 hr; p < 0.001, 60–64 hr; MOVTIM: p = 0.014, 60–64 hr; TOTDIST: p = 0.036, 36–40 hr; p = 0.023, 60–64 hr; MOVNO: p = 0.006, 60–64 hr, Supplemental Tables 1 and 2, Fig. 2).

### mTBI3 altered response to the change of light

**Figure 3:**
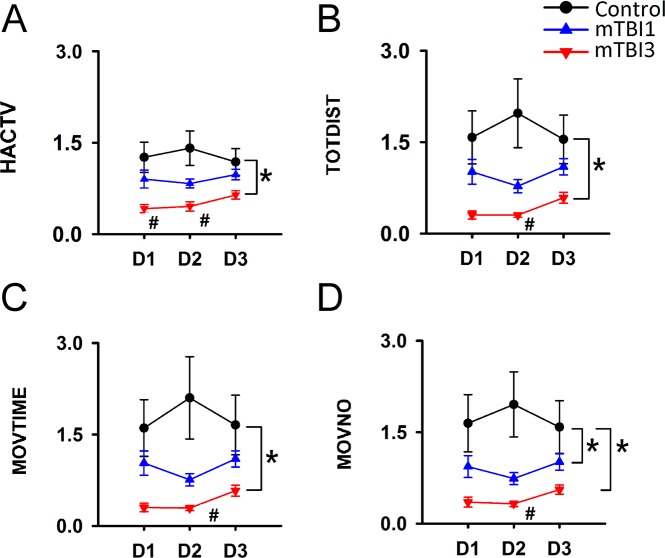
mTBI altered response to the light change. The sensitivity to light was measured by comparing the locomotor activity after and before the light was turned on (i.e., 4-hr-after light on/4-hr-before light on). mTBI3 significantly increased the sensitivity to light (**A**. HACTV, p = 0.001; **B**, Totdist, p = 0.003; **C**, movetime, p = 0.007; **D**, moveno, p = 0.003, Two-way ANOVA).

mTBI3 mice demonstrated an early onset of immobilization in the light cycle (20–24 hr, 44–48 hr, and 68–72 hrs after mTBI, Fig. 2A–D). The response to the change of light was indirectly measured by comparing the locomotor activity after and before the light was turned on (i.e., 4-hr-after light on/4-hr-before light on, Fig. 3). mTBI3 significantly reduced the light/dark activity compared to control (HACTV, p < 0.001; TOTDIST, p = 0.003; MOVTIME, p = 0.007; MOVENO, p = 0.003, two way ANOVA, Fig. 3). No significant difference was found between mTBI1 and control (HACTV, p = 0.076; TOTDIST, p = 0.062; MOVTIME, p = 0.073, two way ANOVA). There is a linear correlation between the number of impacts and light/dark activity (HACTV, p = 0.017; TOTDIST, p = 0.038; MOVTIME, p = 0.055; MOVNO, p = 0.04, see Table 1). These data suggest that mTBI dose-dependently increases response to light change (i.e. mTBI3 > mTBI1 > no-mTBI).

### mTBI altered IBA1, BAX and iNOS activity in the lesioned cortex

**Figure 4:**
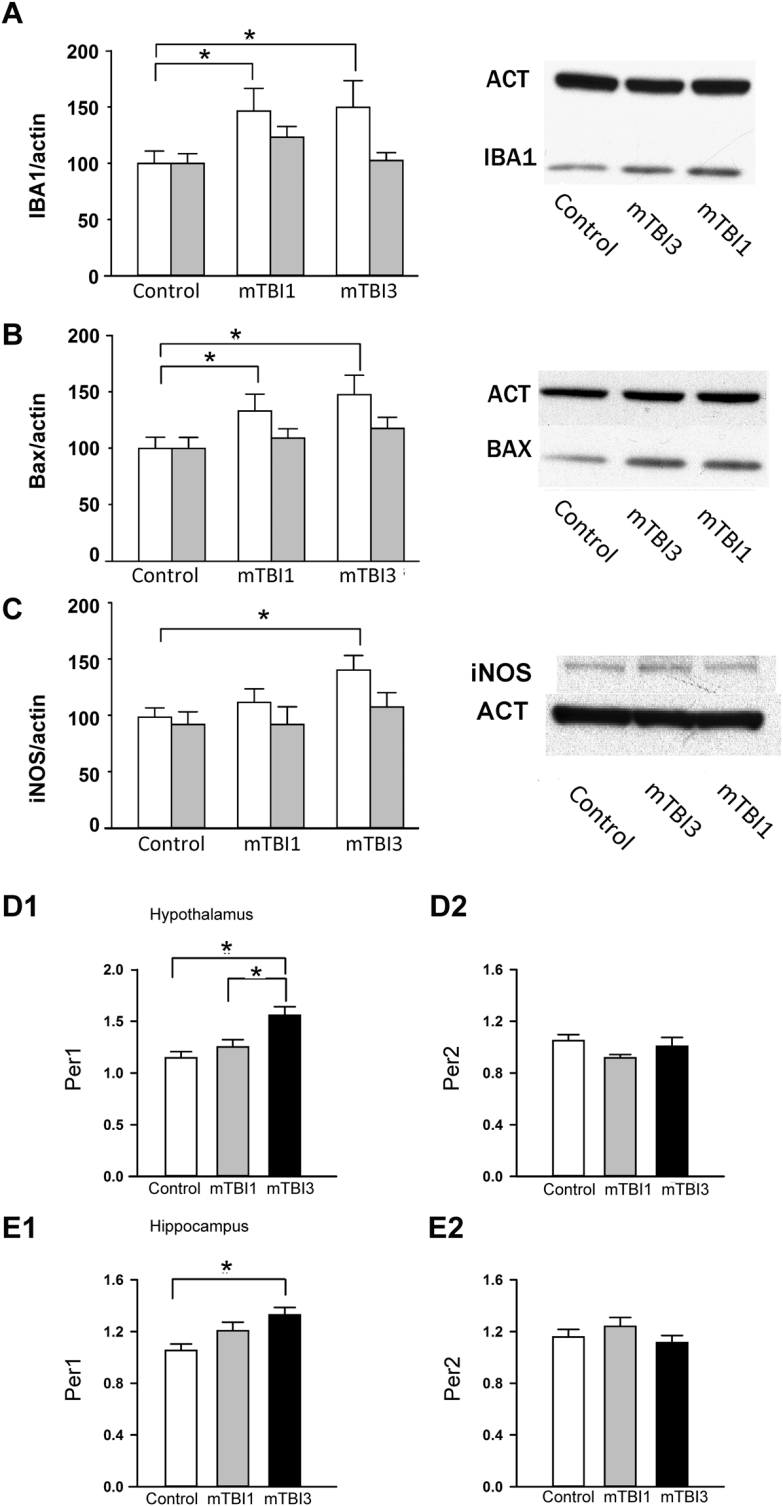
Multiple mTBI increased IBA1, BAX, iNOS immunoreactivity in the lesioned cortex and increased PER1 mRNA expression in hypothalamus and hippocampus. (**A,B**) Brain tissue was collected at 48 hr after the last mTBI. (**A**) In the lesioned side cortex (open bars), mTBI1 and mTBI3 significantly increased IBA1 protein expression. No difference was found on the contralateral side cortex (gray bars). (**B**) mTBI1 and mTBI3 significantly increased BAX expression on the lesioned cortex. (**C**) mTBI3 significantly increased iNOS expression in the lesioned side cortex. (**D,E**) Brain tissues were collected for qRT-PCR analysis at 28 hr after the last mTBI. mTBI3 significantly upregulated Per1 expression In hypothalamus (**D1**) and hippocampus (**E1**). Per2 expression was not altered by mTBI1 or mTBI2 in hypothalamus (**D2**) and hippocampus (**E2**). The expression of target genes (with a modified delta-delta-Ct algorithm) Per1 and Per2 were calculated relative to the endogenous reference gene (Beta-actin and GAPDH average). *p < 0.05, ANOVA. The full length blots for western blot pictures shown in panels (A–C) are presented in Supplemental Fig. S1.

Cerebral cortices were collected from 18 mice (control = 8, mTBI1 = 5, mTBI3 = 5) at 48 hr after mTBI. Both mTBI1 and mTBI3 significantly increased IBA1 expression in the lesioned (right) side cortex (Fig. 4A, p = 0.029, F_2,30_ = 3,977, 2-way ANOVA). Posthoc test indicated a significant elevation of IBA1 in the right cortex after mTBI1 (p = 0.020) or mTBI3 (p = 0.033) compared to control. No difference was found between mTBI1 and mTBI3 (p = 0.869). mTBI1 and mTBI3 significantly increased BAX expression in the lesioned side cortex (Fig. 4B, p = 0.019, F_2,30_ = 4.568, 2-way ANOVA; p = 0.015 mTBI3 vs control, p = 0.046 mTBI1 vs control). mTBI3 also significantly increased iNOS expression in the lesioned side cortex (Fig. 4C, p = 0.041, mTBI3 vs no mTBI control).

### mTBI3 selectively increased PER1 expression

Brain tissues were collected from 31 mice for qRT-PCR analysis at 28 hr after the last mTBI (mTBI1,n = 10; mTBI3, n = 10; and control, n = 11). mTBI3 significantly upregulated Per1 expression in hypothalamus compared to control (Fig. 4D1, p < 0.001, F_2,28_ = 9.288, 1-way ANOVA; p < 0.001) or mTBI1 (p = 0.005). There was no difference between mTBI1 and control (p = 0.289). mTBI1 or mTBI2 did not alter the expression of Per2 (p = 0.162, F_2,28_ = 1.941, 1-way ANOVA, Fig. 4D2). A similar change in Per1 and Per2 expression was found in the hippocampus (Fig. 4E). mTBI3 significantly upregulated Per1 (p = 0.005, F_2,29_ = 6.470, 1-way ANOVA; p = 0.004, vs. no-mTBI), but not Per2 (p = 0.330, Fig. 4E2) in hippocampus.

## Discussion

The principal finding of the present study was that multiple mTBIs preferentially altered the locomotor activity following changes from the light to the dark phase of the daily light-dark cycle. The largest change in activity was found during the 4-hr after the change from dark to light. As we previously reported, the movement in the first 4 hr in the activity chamber was mainly affected by the novel environment to the animals and represented exploratory behavior^28^. mTBI significantly reduced overall horizontal activity, movement time, total distance traveled and movement number during the exploratory phase (0–4 hr) after injury. There were no differences between single and multiple mTBIs. Similar to the acute phase, single or multiple mTBIs suppressed overall locomotor activity in the subacute phase from 4 to 72 hrs after injury. The reduction in locomotor activity mainly occurred in the dark cycle. Dark cycle locomotor activity did not significantly correlate with the number of impacts. Our data suggest that single or multiple mTBIs impaired locomotor function; multiple mTBIs did not dose (# of impacts) -dependently reduce locomotor activity.

In the present study, minimal TBI was induced in isoflurane-anesthetized mice by using a previously characterized weight drop model to deliver transient sheer force to right temporal region without damaging the dura, skull or scalp. Previous studies have indicated that the non-invasive closed-head weight-drop mTBI model causes a long-lasting behavioral impairment in mice in the absence of neurological damage, brain edema or anatomical changes to the brain as observed by MRI imaging^26^. We also reported that no prominent pathological findings were found by TTC staining after mTBI induced by a 30-g weight drop^10^. Our mTBI animal model thus closely mimics clinical cases, which show no primary tissue damage but post-concussive syndromes.

mTBI -mediated behavioral deficits were associated with apoptosis and neuroinflammation^9,10^. We previously demonstrated that the expression of inflammatory genes changed in response to a weight-drop traumatic brain injury^9^. At three days post-injury, upregulated transcripts encoded products marking reactive astrocytes, phagocytes, microglia, and immune-reactive cells were found in the neocortex and hippocampus on the lesioned side brain. In this study, we further demonstrated that IBA1 and BAX immunoreactivities were upregulated at 2 days after the last mTBI in the lesioned side cerebral cortex. As seen in the behavioral response, mTBIs did not dose (# of impacts) -dependently increase IBA1 and BAX expression. These data suggest that increasing the number of mTBIs did not further potentiate these inflammation or apoptosis markers in the lesioned brain and enhance behavioral deficits. Similar findings have been reported in animal models of stroke and TBI such that an earlier minor insult triggers protection against subsequent brain injury^29–31^. This “pre-conditioning” reaction may support minor behavioral and biochemical improvements after multiple mTBI.

Previous studies have indicated that TBI induced a time-dependent inducible nitric oxide synthase (iNOS) expression in the lesioned brain^32–34^. The induction of iNOS paralleled the expression of nitrosative damage marker 3-nitrotyrosine (3NT)^32^. Treatment with an iNOS inhibitor attenuated TUNEL and LTP impairment in the lesioned brain and improved behavioral function after TBI^33–35^. Similarly, suppression of iNOS by antisense RNA improved cytochrome C activity and normalized ATP level in TBI rats^36^. These data suggest that iNOS expression is associated with neurodegenerative changes after TBI. In this study, a significant increase in iNOS immunoreactivity was found in the lesioned cortex at 2 days after mTBI3.

Sleep disturbances are often found in TBI patients^13,37^. The severity and frequency of sleep disturbances can be further enhanced after multiple mTBI^25^. We demonstrated an early onset of immobilization to light after mTBI3 in the light cycle. The sensitivity to light, measured by the light/dark activity ratio, was dose-dependently increased by the number of mTBI (i.e., mTBI3 > mTBI1 > control). The disturbance of diurnal locomotor activity was further illustrated by hyperactivity following initial immobilization in the light cycle in the mTBI3 mice, a symptom similar to insomnia (e.g., waking up during the night) in patients. Our findings provide a clinically relevant experimental model. Previous reports indicated that brain injury upregulated per1 mRNA in the hippocampus^38^. Overexpression of Per1 in transgenic mice lengthened circadian periods, impaired rhythms of locomotor activity, and altered sensitivity to light^39^. Similarly, we found that the locomotor disturbances in the light cycle were associated with dysregulation of the central circadian signals as multiple mTBI selectively upregulated Per1 in hypothalamus and hippocampus. Interestingly, one case study showed that an adolescent male athlete developed multiple symptoms including sleep disturbance, associated with low plasma cortisol level, after the 4^th^ head trauma over 4 months^40^. Repeated blast injury, given 3 times within 30 min, significantly altered serotonin level in plasma, platelets and frontal cortex in mice^41^. The association of sleep disturbance and the expression of these sleep-related transmitters or hormones after repeated mTBI requires further investigations.

The change in locomotor activity in the light/dark cycle in this study may indirectly imply sleep disturbance after mTBI. A similar approach was used to non-invasively evaluate sleep disturbance after a close-head mTBI in mice in other studies^42^. However, the locomotor activity cannot directly translate to sleep and can not differentiate between rapid eye movement (REM) and non-REM sleep. Direct measurement of sleep should involve EMG/EEG^43^ and are required for future validation. As mentioned by Skopin *et al*., implantation of EEG electrodes is invasive and may interfere with the close-head injury^42^.

Cognitive impairment is a common consequence after traumatic brain injury. Multiple mTBI can induce other behavioral deficits. For example, repeated mTBI impaired spatial learning in the Morris water maze in mice^44,45^. We also found that mTBI3 increased depression-like behavior in a forced swimming test (Supplemental Fig. 2). The mechanisms involved in cognitive impairment after multiple mTBI require further investigation.

## Conclusion

We found that single or multiple mTBIs equally suppressed exploratory locomotor activity while they increased brain inflammation and apoptosis. Multiple mTBIs caused mild locomotor improvement, increased disturbances in circadian locomotor activity, and elevated Per1 expression. It is possible that the mild recovery in locomotor activity may obscure the progress of psychological conditions, such as hypersensitivity to light or quality of sleep, after multiple mTBI in patients, athletics, or soldiers.

## Materials and Methods

### Animals and mTBI

Adult male ICR mice (3 months old) were purchased from the BioLASCO, Taiwan and were used for this study. The use of animals was approved by the Animal Care and Use Committee of either the National Health Research Institute, Miaoli, Taiwan (approved number 105080-AC1-E1). All animal experiments complied with the Animal Research: Reporting of *In Vivo* Experiments (ARRIVE) guidelines and were carried out in accordance with the National Institutes of Health guide for the care and use of Laboratory Animals (NIH Publications No. 8023, revised 1978). Animals were housed in a 12-hr dark (6 pm to 6 am) and 12-hr light (6 am to 6 pm) cycle. Animals were anesthetized with isoflurane and were laid on their side. mTBI was conducted by dropping a 30 g metal weight from 80 cm height onto the right temporal skull, anterior to the right ear as previously described^9,10^. A sponge immobilization pad (L:4–5 in; W: 2.7 in; H: 1.8 in) was employed; this allows head movements during the injury. For multiple mTBI, animals received mTBI every week for up to 3 weeks. Animals in control groups were anesthetized for the same duration but were not injured.

### Locomotor behavioral measurement

Locomotion was measured using an infrared activity monitor (Accuscan, Columbus, OH). The open-field motor behavior was examined only once in each control or mTBI animal after the last impact. Mice were individually placed in a 42 × 42 × 21 cm closed plexiglass box which contained horizontal infrared sensors placed 2.5 cm apart. Food and water was available *ad libitum*. The following variables were measured: (i) horizontal activity (HACTV, the total number of beam interruptions that occurred in the horizontal sensors), (ii) movements time (MOVTIME), and (iii) total distance traveled (TOTDIST, the distance, in centimeters, traveled by the animals), (iv) number of movements (MOVNO).

### Western blotting

IBA1, BAX, and iNOS immunoreactivity was examined by Western analysis. Animals were sacrificed by decapitation. The brains were removed and placed in a brain matrix. The right and left frontal cortex were collected and frozen. For Western blotting, the tissue was homogenized in RIPA lysis buffer (Cell Signal). The homogenate was centrifuged at 13200 rpm for 10 min at 4 °C, and the supernatant was collected. A bicinchoninic acid (BCA) protein assay was performed using bovine serum albumin to determine protein concentrations. The samples were diluted with RIPA buffer according to the BCA protein assay. Gels were transferred to a PVDF membrane after electrophoresis. The membranes were blocked in 5% milk at room temp for 1 h. The blots were then probed with primary antibodies against ionized calcium-binding adapter molecule 1 (IBA1, 1: 500, Wako), BCL2-associated X protein (Bax, 1:1000, Santa Cruz), inducible nitric oxide synthase (iNOS,1:1000, BD), or actin (1:10000, Novus) at 4 °C for overnight. The membrane was then incubated with a horseradish peroxidase (HRP)-conjugated secondary antibody (Jackson lab) at room temp for 1 h, followed by washing with 0.1% Tween-20 (in PBS) three times for 10 min each. The light emission signal of the target proteins on the PVDF membrane was generated by using a Western Lightning Plus-ECL (PerkinElmer) and then detected by X-ray film (Cat. No. NEF596, Kodak). The amount of IBA1, Bax and iNOS was normalized with actin on the same membrane. Band intensity was quantified using Image J.

### Quantitative Reverse transcription polymerase chain reaction (qRTPCR)

Brain tissues were collected from 31 mice for qRT-PCR analysis at 28 hr after the last mTBI as previously described^46^. Total RNAs were isolated from using TRIzol Reagent (ThermoFisher, #15596–018) and cDNAs were synthesized from 1 ug total RNA using RevertAid H Minus First Strand cDNA Synthesis Kit (Thermo Scientific, #K1631). The TaqMan Gene Expression Assays primer for specifically detecting mouse Beta-actin (#Mm02619580_g1) and GAPDH (#Mm99999915_g1) were purchased from Thermo Scientific. Quantitative Real-Time PCR (qRT-PCR) was carried out using TaqMan Fast Advanced Master Mix (Life Technologies, #4444557) and Applied Biosystems 7500 Fast Real-Time PCR System. Per1 and Per2 mRNA expression was measured by using SYBR (Luminaris Color HiGreen Low ROX qPCR Master Mix; ThermoScientific). The primers used were as follows: Per1 (GenBank Acc.), forward: 5′-CAGCCGTGCTGCCTACTCATT-3′ and reverse: 5′-AGAGG- CAGCTTGGTGTGTGTC-3′; Per2 (GenBank Acc.), forward: 5′-TGGTCTGGACTGCACATCTGG-3′, reverse: 5′-AGGTCACTTGACGTG- GAGATGG-3′. Expression and normalization of the target genes, Per1 and Per2, were calculated relative to the endogenous reference gene (Beta-actin and GAPDH average) with a modified delta-delta-Ct algorithm that takes gene-specific amplification efficiency into account for accurate calculation. All experiments were duplicated.

### Statistical analysis

All data were expressed as means ± SEM. Behavioral and biochemical data were analyzed using a linear regression, one or two way ANOVA, and post-hoc Fisher LSD test. All analyses were calculated by Sigmaplot software ver 10.0. Statistical significance was defined as p < 0.05.

## Supplementary information

supplementary Figures and Tables
