# Supplementary material for: Repetitive mild traumatic brain injury alters diurnal locomotor activity and response to the light change in mice
Source: Sci Rep. 2019 Oct 1;9:14067. doi: 10.1038/s41598-019-50513-5 (PMC6773703; doi:10.1038/s41598-019-50513-5)
Supplement: Supplementary file 1 — supplementary Figures and Tables [file 41598_2019_50513_MOESM1_ESM.pdf]

Supplemental Title page

Manuscript title: Repetitive mild traumatic brain injury alters diurnal locomotor activity and response to the light change in mice

Authors: Yu-Syuan Wang,[1] Wei Hsieh,[1] Jia-Ru Chung,[1] Tsuo-Hung Lan,[1,2,3] Yun Wang[1,*]

[1]Center for Neuropsychiatric Research, National Health Research Institutes, Taiwan

[2]Department of Psychiatry, Taichung Veteran General Hospital, Taiwan

[3]Department of Psychiatry, School of Medicine, National Yang-Ming University, Taiwan

Supplemental Figure 1

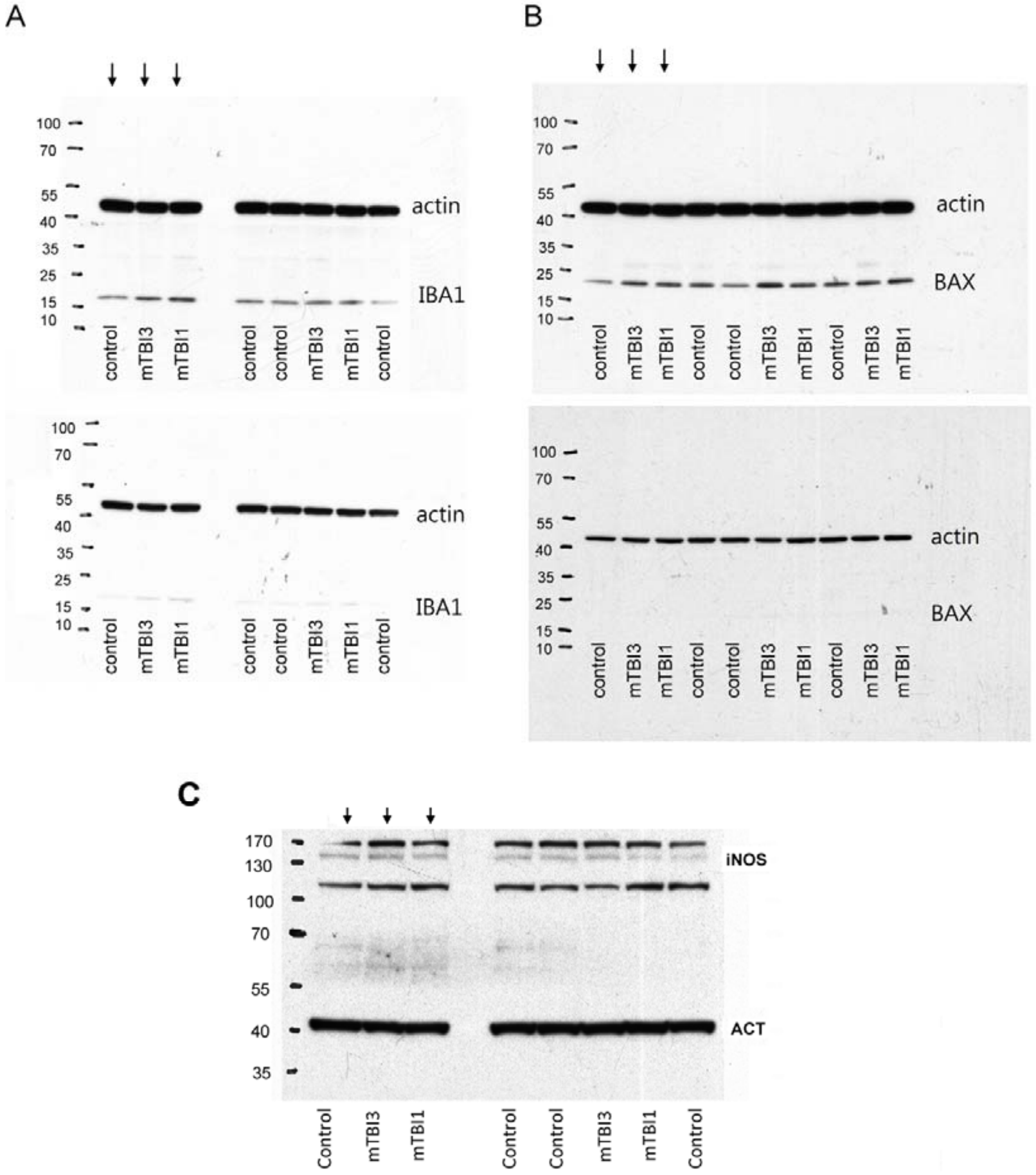

The full-length western blots of gels of IBA1, BAX, iNOS, and β-actin presented in Fig 4 A-C of the main article. The same films were examined at two exposure time to determine the immunoreactivity of IBA1 and BAX (A and B, top panels) and β-actin (A and B, lower panels). Arrows represent the lanes selected for Fig 4 A-C.

Supplemental Figure 2

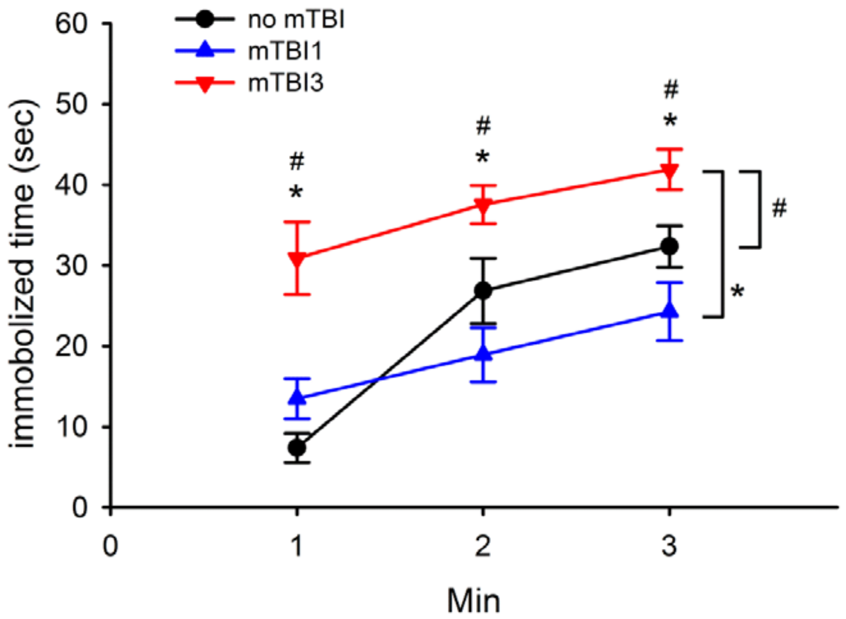

Multiple mTBI increased depression–like behavior in a forced swimming test. Mice were separated to three groups (no mTBI n=16, mTBI1 n=8, mTBI3, n=8). Forced swimming test was conducted at one day after the last mTBI or sham control procedures. Animals were placed individually in a clear plexiglass cylinder (20 cm high x 8 cm diameter) filled with tap water (15 cm) at 25 ± 1 °C. The movements of the mice were recorded by a camera for 6-min. The duration (seconds) of immobility in the first 3-min was analyzed through EthoVision XT 7 software by an investigator blinded to the experimental groups. mTBI3 significantly increased the immobilized time (mTBI3 vs mTBI1, $p<0.001$; mTBI3 vs no mTBIm $p<0.001$, 2-Way ANOVA). There is no significant difference between mTBI1 and no mTBI ($p=0.216$). [#]Significant difference between mTBI3 and no mTBI. *Significant difference between mTBI3 and mTBI1.

Supplemental Table 1: mTBI1 and mTBI3 altered diurnal horizontal activity (HACTV).

HACTV	no mTBI	(n=20)	mTBI1	(n=8)	mTBI3	(n=8)	*p value		
time (hour)	mean	± sem	mean	± sem	mean	± sem	mTBI3 vs mTBI1	mTBI3 vs no mTBI	mTBI1 vs no mTBI
4-8	9985.4	749.8	7992.0	1237.0	17637.6	1323.3	0.013	0.019	0.54
8-12	26488.9	2791.5	15895.4	1758.8	20939.9	3290.7	0.194	0.088	0.001
12-16	29768.4	2636.0	19641.9	2331.8	20867.0	2078.9	0.752	0.006	0.002
16-20	25325.7	2802.7	24812.4	2545.4	26195.1	3731.9	0.722	0.789	0.874
20-24	21641.6	1843.9	20612.6	2344.6	10131.1	1387.7	0.007	<0.001	0.751
24-28	6790.4	556.0	8652.9	1342.9	14753.9	2395.8	0.117	0.014	0.567
28-32	8472.1	640.1	5060.5	327.7	13339.4	1502.8	0.033	0.134	0.294
32-36	22560.6	1888.2	13457.9	909.4	19081.4	1401.4	0.148	0.284	0.005
36-40	32261.4	2667.0	15770.9	1454.9	27535.3	3002.6	0.003	0.146	<0.001
40-44	22473.4	2648.9	19674.8	1964.5	24269.0	2514.3	0.237	0.581	0.389
44-48	21337.9	1578.5	16219.5	1855.9	10131.1	1387.7	0.08	<0.001	0.116
48-52	5579.8	587.9	8123.6	1037.1	14753.9	2395.8	0.233	0.027	0.434
52-56	9252.4	580.1	6329.6	582.9	13339.4	1502.8	0.165	0.447	0.368
56-60	23455.3	2245.3	13010.6	1064.1	17535.0	626.5	0.244	0.069	0.001
60-64	27779.5	2169.8	14628.8	2129.5	28250.5	2815.7	<0.001	0.885	<0.001
64-68	23621.0	2491.2	18098.1	2764.7	23431.3	3234.5	0.17	0.953	0.09
68-72	19853.5	1858.5	17228.0	2610.0	14537.4	2539.5	0.488	0.102	0.419

*Two way ANOVA + Fisher LSD test

Supplemental Table 2: mTBI1 and mTBI3 altered diurnal movement time (MOVETIM).

MOVETIME	no mTBI (n=20)		mTBI1 (n=8)		mTBI3 (n=8)		*p value		
time (hour)	mean	± sem	mean	± sem	mean	± sem	mTBI3 vs mTBI1	mTBI3 vs no mTBI	mTBI1 vs no mTBI
4-8	306.7	32.1	298.9	209.3	584.4	34.7	0.082	0.043	0.954
8-12	974.7	121.0	598.1	251.2	695.9	123.1	0.551	0.042	0.006
12-16	1034.4	101.2	789.3	377.0	574.5	59.3	0.19	<0.001	0.074
16-20	901.9	116.6	895.8	426.6	847.0	137.4	0.766	0.689	0.964
20-24	758.6	80.0	804.6	400.1	231.7	43.5	<0.001	<0.001	0.737
24-28	156.6	23.5	255.0	152.1	391.7	84.9	0.404	0.087	0.473
28-32	177.7	23.7	92.4	42.6	285.3	62.6	0.239	0.433	0.534
32-36	656.0	70.3	396.7	126.7	484.0	45.3	0.210	0.594	0.059
36-40	1015.4	119.1	534.1	281.5	791.4	140.6	0.117	0.103	<0.001
40-44	690.6	100.7	713.6	268.0	708.6	95.1	0.976	0.895	0.867
44-48	671.8	61.5	551.2	257.7	185.0	24.5	0.026	<0.001	0.38
48-52	104.1	18.5	233.0	123.7	308.3	47.8	0.646	0.137	0.347
52-56	182.2	24.4	115.4	79.0	241.8	62.1	0.441	0.664	0.626
56-60	685.5	92.1	339.0	160.7	406.3	29.7	0.681	0.042	0.012
60-64	827.8	99.1	393.9	237.0	799.3	110.6	0.014	0.835	0.002
64-68	756.2	105.1	576.0	327.6	698.7	144.2	0.454	0.675	0.189
68-72	632.6	83.1	592.3	332.9	389.7	98.3	0.217	0.077	0.769

*Two way ANOVA + Fisher LSD test
